# A smartphone-read ultrasensitive and quantitative saliva test for COVID-19

**DOI:** 10.1126/sciadv.abe3703

**Published:** 2021-01-08

**Authors:** Bo Ning, Tao Yu, Shengwei Zhang, Zhen Huang, Di Tian, Zhen Lin, Alex Niu, Nadia Golden, Krystle Hensley, Breanna Threeton, Christopher J. Lyon, Xiao-Ming Yin, Chad J. Roy, Nakhle S. Saba, Jay Rappaport, Qingshan Wei, Tony Y. Hu

**Affiliations:** 1 Center for Cellular and Molecular Diagnostics, Tulane University School of Medicine, 1430 Tulane Ave., New Orleans, LA 70112, USA.; 2 Department of Biochemistry and Molecular Biology, Tulane University School of Medicine, 1430 Tulane Ave., New Orleans, LA 70112, USA.; 3 Department of Chemical and Biomolecular Engineering, North Carolina State University, Raleigh, NC 27606, USA.; 4 State Key Laboratory of Food Science and Technology, Nanchang University, Nanchang 330047, China.; 5 Department of Pathology and Laboratory Medicine, Tulane University School of Medicine, 1430 Tulane Ave., New Orleans, LA 70112, USA.; 6 Section of Hematology and Medical Oncology, Deming Department of Medicine, Tulane University, New Orleans, LA 70112, USA.; 7 Tuberculosis Research Performance Core, Tulane National Primate Research Center, Covington, LA 70433, USA.; 8 High Containment Research Performance Core, Tulane National Primate Research Center, Covington, LA 70433, USA.; 9 Tulane School of Medicine, Tulane National Primate Research Center, New Orleans, LA 70112, USA.

## Abstract

Point-of-care COVID-19 assays that are more sensitive than the current RT-PCR (reverse transcription polymerase chain reaction) gold standard assay are needed to improve disease control efforts. We describe the development of a portable, ultrasensitive saliva-based COVID-19 assay with a 15-min sample-to-answer time that does not require RNA isolation or laboratory equipment. This assay uses CRISPR-Cas12a activity to enhance viral amplicon signal, which is stimulated by the laser diode of a smartphone-based fluorescence microscope device. This device robustly quantified viral load over a broad linear range (1 to 10^5^ copies/μl) and exhibited a limit of detection (0.38 copies/μl) below that of the RT-PCR reference assay. CRISPR-read SARS-CoV-2 (severe acute respiratory syndrome coronavirus 2) RNA levels were similar in patient saliva and nasal swabs, and viral loads measured by RT-PCR and the smartphone-read CRISPR assay demonstrated good correlation, supporting the potential use of this portable assay for saliva-based point-of-care COVID-19 diagnosis.

## Teaser

An isolation-free smartphone-based saliva assay can diagnose COVID-19 cases and quantify viral load within 15 min.

## INTRODUCTION

SARS-CoV-2 (severe acute respiratory syndrome coronavirus 2) has rapidly spread from its initial outbreak site to produce a pandemic (*1*) that has caused >674,000 deaths within its first 7 months (*2*), but accurate, sensitive, and large-scale testing for SARS-CoV-2 still presents a challenge for ongoing disease control efforts. Future assays required to expand COVID-19 testing capacity should (i) use samples that can be easily collected, (ii) have greater sensitivity than the current reference standard and allow viral quantification for treatment monitoring, and (iii) require minimal training and equipment to obtain valid, robust, and quantitative results from samples containing a broad range of virus concentrations.

Most COVID-19 assays require nasopharyngeal swab samples that must be collected in airborne infection isolation rooms by medical professionals wearing full protective gear. Nasal swab sample collection procedures may be better tolerated than those for nasopharyngeal swab samples, but it is not clear if nasal swab collection involves less transmission risk. Also, it is not clear if these samples are comparable to nasopharyngeal swabs, as small studies have reported lower detection rates for respiratory viruses analyzed by nucleic acid amplification (NAA) of nasal versus nasopharyngeal swab samples (*3*, *4*). However, recent studies indicate that saliva and nasopharyngeal SARS-CoV-2 results exhibit correlation during early infection, and development of saliva-based COVID-19 assays could reduce or eliminate the involvement of medical personnel in sample collection, because saliva collection would not require special materials, training, or infrastructure.

Saliva samples offer practical and logistical advantages for diagnostic and screening efforts, because they can be directly collected by the patient, reducing the need for, and exposure risk of, medical personnel. Saliva samples also do not need to be collected in airborne infection isolation rooms, which allows collections in outpatient clinics, community testing sites, or other locations as needed to meet local needs. Both these features could improve potential testing bottlenecks and enhance diagnostic and screening efforts by reducing the labor required from medical professionals.

Quantitative reverse transcriptase polymerase chain reaction (RT-qPCR) is the primary means used to diagnose COVID-19 using respiratory samples and saliva (*5*, *6*). However, RT-qPCR has several drawbacks for efforts to expand screening capacity: It requires technical expertise and expensive equipment, exhibits a notable false-negative rate, and requires that trained personnel wear extensive personal protective equipment to safely collect valid diagnostic samples. New rapid nucleic acid tests, such as the Abbott ID Now system, do not require the technical expertise or sophisticated equipment necessary for RT-qPCR and have the potential to expand COVID-19 diagnosis capacity outside traditional clinical laboratory settings. Unfortunately, the reported positive percent agreement (PPA) rate of the Abbott ID Now system with RT-qPCR (73.9 to 80.4%) is unacceptable due to its alarming false-negative rate (*7*–*9*).

New NAA strategies may, however, reduce the infrastructure and expertise required to obtain ultrasensitive diagnostic results. Isothermal NAA approaches, such as recombinase polymerase amplification (RPA) or loop-mediated isothermal amplification (LAMP), can reduce equipment demands, while CRISPR-Cas activity can be used to enhance detection sensitivity (*10*–*18*). Several groups have now reported COVID-19 diagnostic assays that use RT-RPA or RT-LAMP, with or without CRISPR-mediated target detection (*14*, *19*–*23*), including studies that used colorimetric lateral flow assay formats suitable for use in resource limited settings (*14*, *23*). However, sensitivity limits reported for these assays (5 to 10 copies/μl) are higher than the reported limit of detection for the RT-qPCR reference standard (1 copy/μl).

Here, we report the development of an RT-RPA CRISPR–fluorescence detection system (FDS) assay to sensitively quantify SARS-CoV-2 present in saliva, without RNA isolation, and adapted this assay to a smartphone-read chip format. To create this system, we first optimized the lysis buffer conditions required to reproducibly maximize signal from the subsequent CRISPR-FDS assay. Next, we determined the linear range and LOD of the optimized assay, evaluated the correspondence of CRISPR-FDS and RT-qPCR assay results upon analysis of healthy saliva samples spiked with and without SARS-CoV-2 RNA, and analyzed the correlation between viral RNA load in paired saliva and nasal swab samples obtained from patients diagnosed with COVID-19. These studies were followed by CRISPR-FDS evaluation of the time course of SARS-CoV-2 RNA expression in nasal and pharyngeal swab samples of a nonhuman primate of COVID-19 and a comparison of CRISPR-FDS–quantified viral RNA load in correlation between paired nasal swab and saliva samples obtained from individuals screened for COVID-19. Last, we adapted this CRISPR-FDS laboratory test to a chip format assay read by a prototype smartphone-based fluorescence microscope device designed for point-of-care use and evaluated its analytical performance and the agreement between smartphone-read CRISPR-FDS assay and RT-qPCR results for saliva samples obtained from patients screened for COVID-19. This saliva assay exhibited complete concordance with RT-qPCR results from paired nasopharyngeal samples, a low LOD, and a broad linear range, and thus appears suitable for ultrasensitive point-of-care COVID-19 screening efforts.

## RESULTS

### Optimization of CRISPR-FDS for extraction-free detection of SARS-COV-2 RNA in saliva

**Fig. 1:**
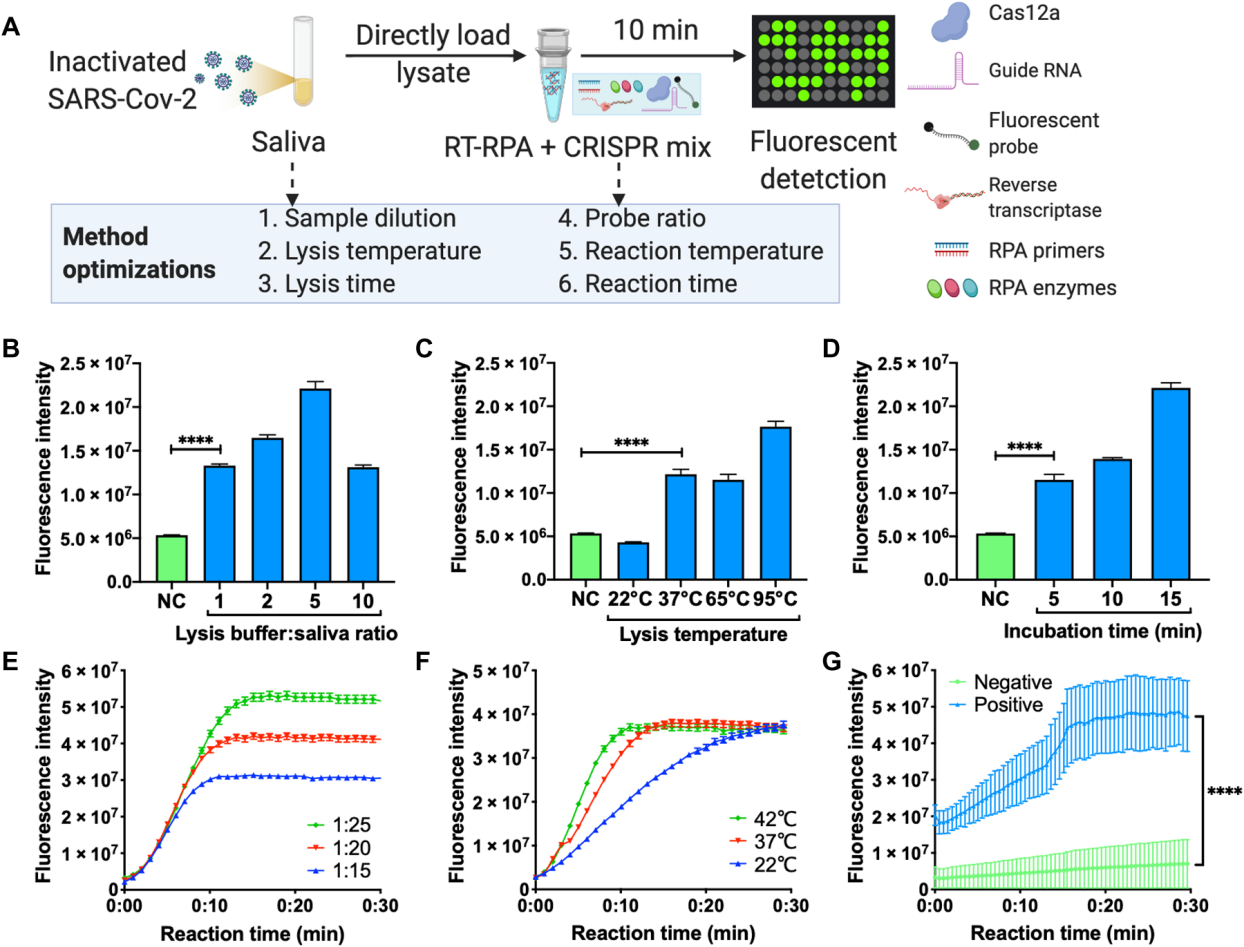
Optimization of an RNA extraction–free assay for saliva-based COVID-19 diagnosis. (**A**) Optimization workflow for RNA recovery and CRISPR-FDS reaction efficiency. Steps 1 to 3: Sample lysis conditions were evaluated by CRISPR-FDS signal produced by healthy donor saliva (10 μl) spiked with heat-inactivated SARS-CoV-2 virus (100 copies/μl) after optimizing: 1. sample dilution with increasing lysis buffer [(**B**) 1:1 to 1:10 saliva to buffer; 65°C for 5 min]; 2. lysis temperature conditions [(**C**) 22° to 95°C; 1:1 dilution for 5 min]; and 3. lysis times [(**D**) 5, 10, or 15 min; 1:1 dilution at 65°C]. NC, negative control [50 μl of human RNA (100 ng/ml)]. Steps 4 to 6: CRISPR reaction efficiency was optimized by 4. titrating the Cas12a/gRNA-to-probe molar ratio [1:15 to 1:25 Cas12a/gRNA to probe; (**E**) 42°C for 30 min]; 5. altering reaction temperature [(**F**) 22° to 42°C; 30-min reaction with a 1:25 Cas12a/gRNA-to-probe ratio); and 6. evaluating the difference in signal from of a representative COVID-19 patient and healthy donor over time [(**G**) 30 min at 42°C with a 1:25 Cas12a/gRNA-to-probe ratio]. Data represent the means ± SD of three technical replicates; ****P < 0.0001.

A saliva-based point-of-care COVID-19 assay should, ideally, function as a single-step assay that can be read without any additional equipment or infrastructure, while retaining high sensitivity (fig. S1). Because CRISPR-FDS has high sensitivity using a simple workflow that requires minimal equipment, we used its assay protocol as a starting approach and optimized aspects of its lysis procedure (sample-to-buffer ratio, and incubation temperature and time) and RT-PCR CRISPR reaction [Cas12/guide RNA(gRNA)–to–probe ratio, and reaction temperature and time] to improve workflow and performance (Fig. 1A).

Most high-sensitivity NAA assays analyze purified RNA samples isolated in multistep procedures that require additional laboratory equipment. This additional step must be eliminated to allow the development of an integrated assay. We thus optimized the viral lysis procedure to determine the conditions that would allow viral lysis samples to be directly analyzed by CRISPR-FDS without a separate isolation step, using a cell lysis procedure compatible with PCR as the base condition. CRISPR-FDS exhibited robust performance when healthy donor saliva samples spiked with SARS-CoV-2 were mixed with increasing lysis buffer volumes (1:1 to 1:10, saliva to buffer) before standard heat denaturation (65°C for 5 min) and direct analysis by CRISPR-FDS (Fig. 1B). SARS-CoV-2 RNA signal increased with lysis buffer concentration up to a 1:5 ratio. However, signals detected at the 1:1 and 1:10 ratios were similar and ~40% less than the signal detected at the 1:5 ratio, indicating that CRISPR-FDS results were not markedly affected by the sample-to-buffer ratio. Given this robust response, we elected to use a 1:1 sample-to-buffer ratio for all further studies.

Because temperature control can be a notable factor in point-of-care assays, we next analyzed the effect of lysis temperature on CRISPR-FDS signal. No signal was detected when lysis reactions were performed at room temperature (~22°C), similar CRISPR-FDS signal was detected at 37° and 65°C, the manufacturer-recommended lysis temperature, with the most signal detected at 95°C (Fig. 1C). We next examined the effect of lysis time on CRISPR-FDS signal when using a 1:1 saliva-to-buffer ratio at 65°C and found that signal increased with incubation time out to the final 15-min time point (Fig. 1D). Given that there was no difference between 65° and 37°C lysis, we elected to use 37°C for the lysis temperature, with the assumption that this minimum temperature would be the simplest to achieve, maintain, and measure in most point-of-care settings, although temperature deviations above this minimum would not negatively influence the lysis reaction. We also chose to use a 5-min lysis time to minimize overall assay performance time.

We next evaluated CRISPR-FDS saliva assay performance at 42°C, the maximum recommended temperature for Cas12a reactions, varying reaction time and Cas12a/gRNA-to-probe ratio to determine the optimal conditions. Increasing probe concentration enhanced CRISPR-FDS signal at 37°C but increased reaction completion time, with 1:20 and 1:25 ratios requiring 20 and 40% longer, respectively, than 1:15 assays, while producing 35 and 70% more signal (Fig. 1E). Reducing the CRISPR-FDS reaction temperature increased assay completion times but did not alter the final signal, with CRISPR-FDS assays performed at 42°, 37°, and 22°C reaching completion after 11, 15, and 27 min of incubation, respectively (Fig. 1F). CRISPR-FDS signals detected after 10 min at 42°, 37°, and 22°C reached 97, 84, and 50% of the signal detected at reaction completion, respectively, allowing robust detection over a broad temperature range at this time point. We next evaluated the specificity of our RT-RPA-CRISPR assay using 39 negative control samples that each contained >10^6^ genome copies/ml of different bacterial/viral/fungal species that can cause respiratory infections, including influenza A and B and respiratory syncytial virus (RSV). No positive results were detected for any of these negative control samples in triplicate assays (table S3). Individual CRISPR-FDS assays could be generated for each of these respiratory infections by replacing the gRNA used in the current assay with gRNAs that recognize gene targets specific for each of these pathogens. Multiplex detection within a signal assay reaction is not feasible, however, because CRISPR-FDS uses the nonspecific single-strand DNA cleavage activity of the bound Cas12a/gRNA-DNA complex to derepress the fluorescent signal from a quenched oligonucleotide probe.

On the basis of these results, we next examined the diagnostic potential of a rapid saliva-based CRISPR-FDS assay in which lysis (5 min; 1:1 sample to buffer) and CRISPR (10 min) incubation steps were performed at 37°C, using a 1:25 CRISPR/gRNA-to-probe ratio. CRISPR-FDS assay results obtained upon saliva from a patient with confirmed COVID-19 and from a healthy individual indicated that this assay can readily distinguish between these individuals (Fig. 1G).

### Evaluation of a saliva-based CRISPR-FDS assay for quantitative COVID-19 diagnosis

**Fig. 2:**
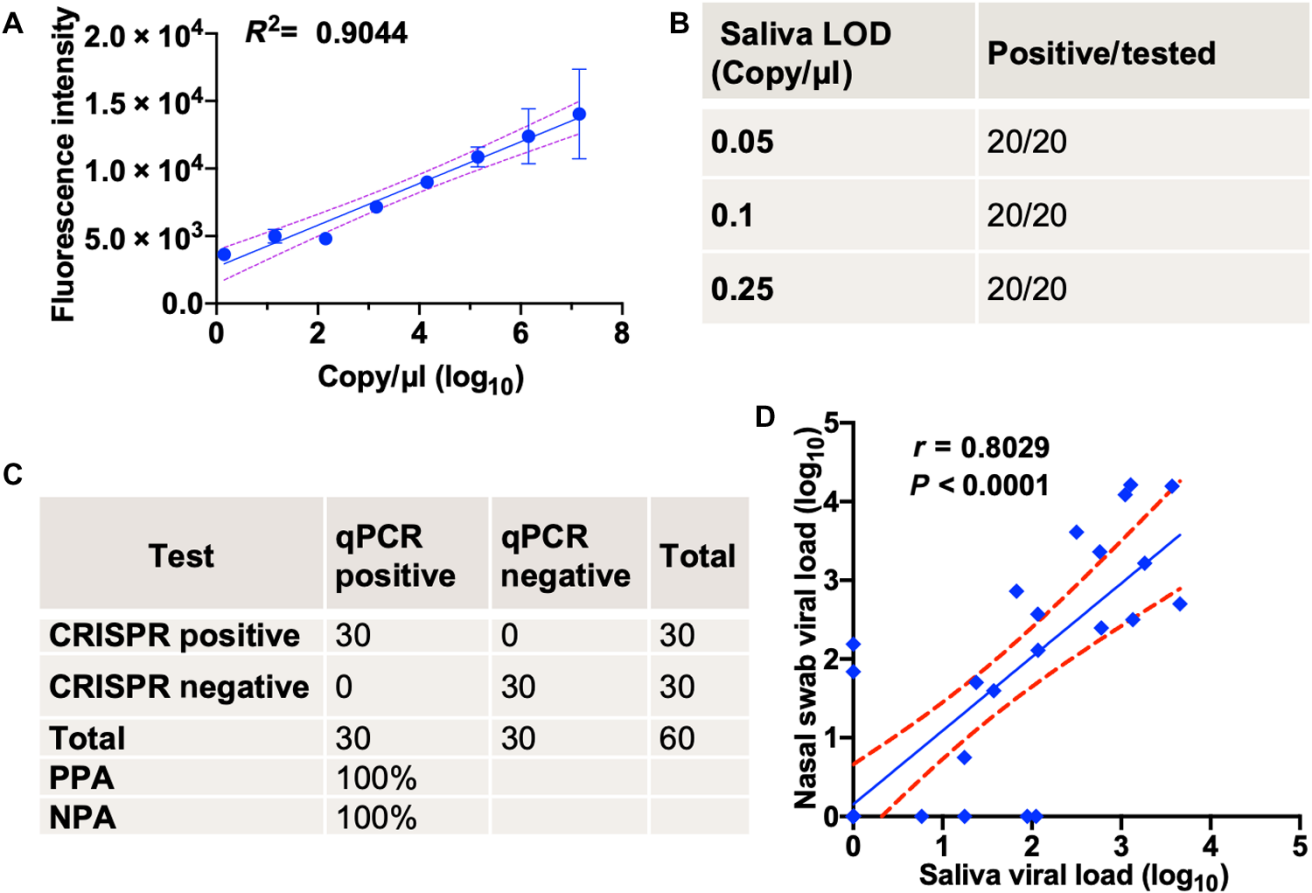
Analytical and clinical validation of the saliva-based CRISPR-FDS assay. (**A**) CRISPR-FDS signal from aliquots of healthy donor saliva spiked with a broad range (1 to 10^7^ copies/μl) of SARS-Cov-2 virus. (**B**) Reproducibility of CRISPR-FDS–positive results in saliva containing low viral loads. (**C**) Correspondence and PPA and negative PA (NPA) of RT-qPCR and CRISPR-FDS results for aliquots of saliva from a healthy individual spiked with or without SARS-CoV-2 virus. (**D**) CRISPR-FDS correlation of paired saliva and nasal swab samples from 31 COVID-19 cases, indicating the linear regression line (solid) and the limits of its 95% CIs (dashed). Data represent the means ± SD of three replicates.

Next, we analyzed the linear range and other properties of the optimized saliva-based CRISPR-FDS COVID-19 assay using a standard curve generated by spiking a known amount of heat-inactivated virus into healthy donor saliva and performing serial dilutions in healthy donor saliva. Results from this analysis demonstrated good linearity (*R*^2^ = 0.90) across a broad range of virus concentrations (1 to 10^7^ copies/μl; Fig. 2A), with an LOD (*24*) of 0.05 copies/μl. Subsequent CRISPR-FDS analysis of 20 replicate samples containing 0.05, 0.1, and 0.25 copies/μl detected positive signal in all samples, suggesting that the actual LOD is <0.05 copies/μl (Fig. 2B). CRISPR-FDS demonstrated complete concordance with RT-qPCR when analyzing saliva aliquots spiked with or without virus, at concentrations matching 1× to 2× the LOD (0.05 and 0.1 copies/μl, respectively) of the CRISP-FDS assay (Fig. 2C). PPA and negative PA (NPA) estimates remained high after adjusting for sample size [100%; 95% confidence interval (CI), 85.8 to 100]. Comparison of CRISPR-FDS results for paired saliva and nasal swab samples obtained from a cohort of 31 individuals screened for COVID-19 (Fig. 2D**)** indicated that viral loads were similar in these samples and demonstrated reasonable correlation (*r* = 0.8029; *P* < 0.0001), and SARS-CoV-2 RNA levels remained stable in saliva stored at 4°C for up to 7 days after collection (fig. S2).

### Saliva virus levels are elevated throughout infection in a rhesus macaque model of COVID-19

**Fig. 3:**
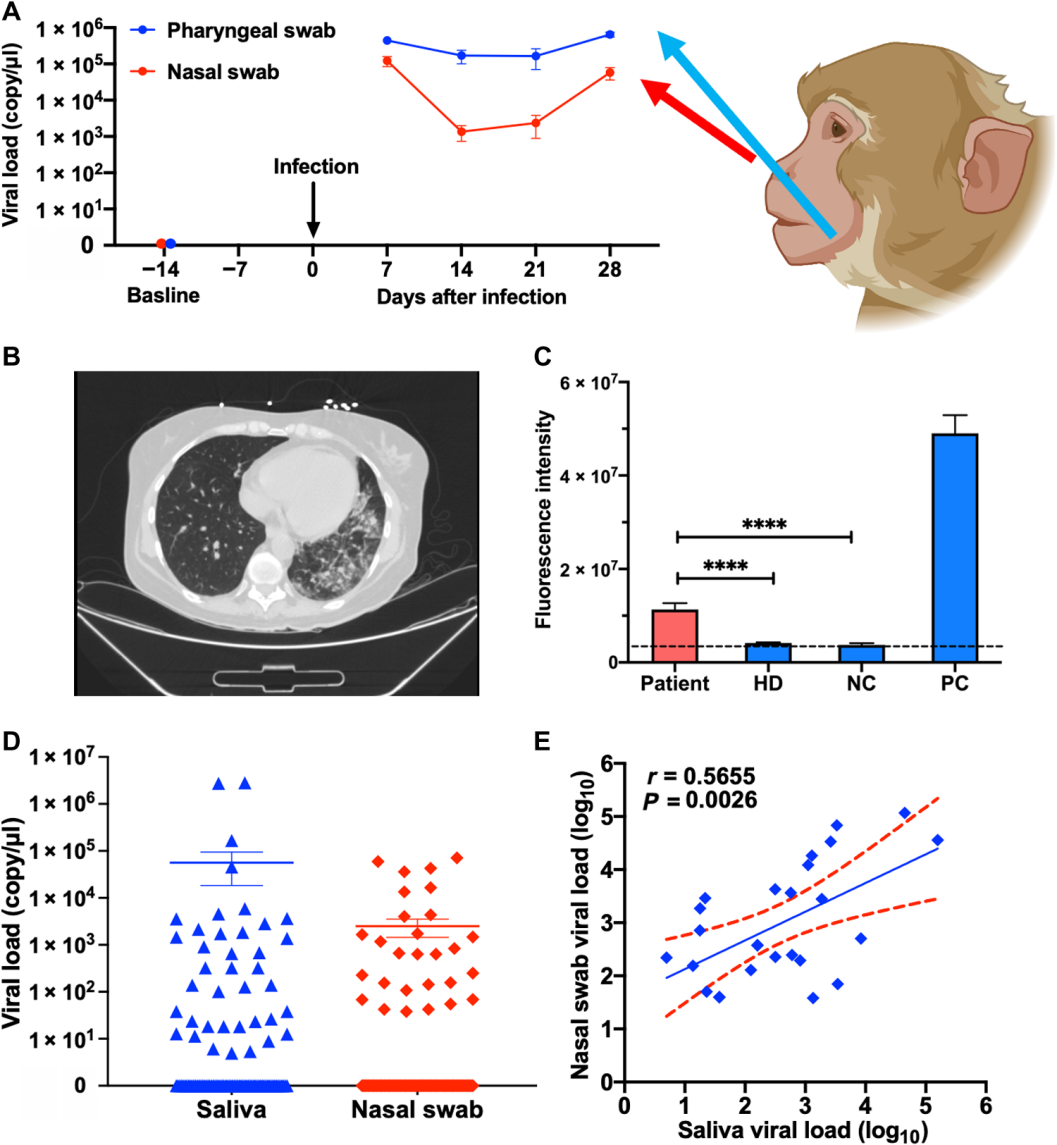
CRISPR-FDS detection of SARS-Cov-2 RNA in animal and patient samples. (**A**) CRISPR-FDS results for RNA isolated from nasal and oropharyngeal (saliva surrogate) swab samples collected from a nonhuman primate model of COVID-19 at baseline (14 days before SARS-CoV-2 infection) and at 7, 14, 21, and 28 days after infection. Data represent the means ± SD of seven animals. (**B**) Chest computed tomography (CT) scan and (**C**) saliva CRISPR-FDS results for a patient who demonstrated pathology consistent with COVID-19 but had two negative nasal swab RT-qPCR results, compared to results from saliva of a healthy donor (HD) with a negative nasal swab RT-qPCR result, and negative control (NC; no template added) and positive control samples (10^2^ copies synthetic SARS-CoV-2 RNA). (**D**) CRISPR-FDS analysis of SARS-CoV-2 viral load distributions in paired saliva and nasal swab samples from 103 individuals undergoing screening for COVID-19. (**E**) CRISPR-FDS correlation of paired saliva and nasal swab samples from 103 COVID-19 cases. Data represent the means ± SD of three replicates.

Saliva from patients with COVID-19 contains SARS-CoV-2 RNA (*25*–*27*), and saliva and nasal samples exhibit high PPA when analyzed within 7 days of COVID-19 symptom onset, which declines thereafter, with an indication that saliva samples may remain positive longer (*28*). Given the uncertainty involved in human exposure events, it is not possible to evaluate the relative diagnostic use of saliva and nasopharyngeal swab samples early in infection. To address this question, we analyzed paired nasal and oropharyngeal (saliva analog) swabs obtained from seven nonhuman primates before and after SARS-CoV-2 infection. CRISPR-FDS determined that mean SARS-CoV-2 RNA levels were 3.6-fold to 124-fold higher, and more stable, in oropharyngeal versus nasal swab samples at all time points after infection (Fig. 3A). These data, and limited human data (*28*), suggest that saliva may represent a more robust diagnostic sample than nasal swabs both early and later in infection.

Anecdotal evidence also suggests that saliva CRISPR-FDS results may have greater diagnostic sensitivity than nasopharyngeal swab RT-qPCR results, as illustrated by a COVID-19 patient whose nasal and nasopharyngeal swabs tested negative by RT-PCR but whose saliva tested positive by CRISPR-FDS (*29*). In this case, a 53-year-old woman with Philadelphia-negative B cell acute lymphoblastic leukemia presented with fever, shortness of breath, cough, and tachycardia. A chest x-ray revealed bilateral lung infiltrates that were confirmed by a chest computed tomography scan that revealed ground glass opacities suggestive of COVID-19 (Fig. 3B). However, the nasal and nasopharyngeal RT-PCR swab tests (Abbott and Roche) conducted as her respiratory status deteriorated to require 4 liter/min oxygen via nasal cannula were negative for SARS-CoV-2. Additional infectious causes were ruled out using the BioFire respiratory panel, serum fungal markers, and serum tests for cytomegalovirus, Epstein-Barr virus, adenovirus, *Legionella*, and *Cryptococcus*. Continued suspicion of COVID-19 led to investigational RT-PCR CRISPR-FDS testing of her saliva, which was determined to be SARS-CoV-2 positive (Fig. 3C). This patient was transferred to the COVID-19 ward for isolation, where she received one unit (200 ml) of COVID-19 convalescent plasma. Within 24 hours, she reported improvements in shortness of breath and cough, exhibited fever resolution, and reduction in her oxygen requirements leading to her eventual discharge without supplemental oxygen. This report, albeit anecdotal, supports the ability of a saliva CRISPR-FDS assay to detect COVID-19 cases missed by RT-PCR assay. In this case, the CRISPR-FDS saliva assay results were instrumental in identifying the false-negative RT-PCR results, which allowed rapid initiation of SARS-CoV-2–directed therapy with convalescent plasma to reverse COVID-19 pathology.

Subsequent CRISPR-FDS analysis of 103 paired saliva and nasal swab samples obtained from individuals screened for COVID-19 detected SARS-CoV-2 RNA in more saliva than nasal swab samples (44 versus 28, Fig. 3D). Viral load was strongly correlated in the 24 individuals with two positive samples (Fig. 3E).

### Design and diagnostic performance of the COVID-19 CRISP-FDS saliva assay read on a chip

These results support the hypothesis that saliva is a valid specimen for COVID-19 diagnosis and can yield results comparable or superior to those obtained with nasopharyngeal or nasal swab samples to reduce the materials, expertise, and infrastructure required to for sample collection. However, this assay still requires a benchtop fluorescence reader for assay evaluation and quantification, limiting its use to sites equipped with such devices. Smartphone-based devices have become an appealing handheld platform to read multiple different assay types (*30*–*34*), and we hypothesized that a smartphone device could similarly allow sensitive and quantitative readout of saliva CRISPR-FDS assays at sites lacking basic laboratory equipment or infrastructure.

Such an approach would require that the CRISPR-FDS assay be reformatted to be easily read by a smartphone camera. To address this issue, we designed and fabricated a proof-of-concept compact assay chip (25 × 35 × 4 mm) suitable for an on-chip CRISPR-FDS saliva assay inserted into a smartphone device functioning as a fluorescence microscope for capture by the field of view (FOV) of its camera. This prototype chip consisted of a layer of polydimethylsiloxane (PDMS) mounted on a glass microscope slide. PDMS was selected for this application because it is a chemically inert and optically clear silicone elastomer that can spontaneously adhere to glass surfaces after plasmonic oxidation, because it allows assay wells to be excited by low-angle laser illumination, and because this inexpensive PDMS/glass format (∼$0.7 per chip) can be readily modified to improve assay functionality.

**Fig. 4:**
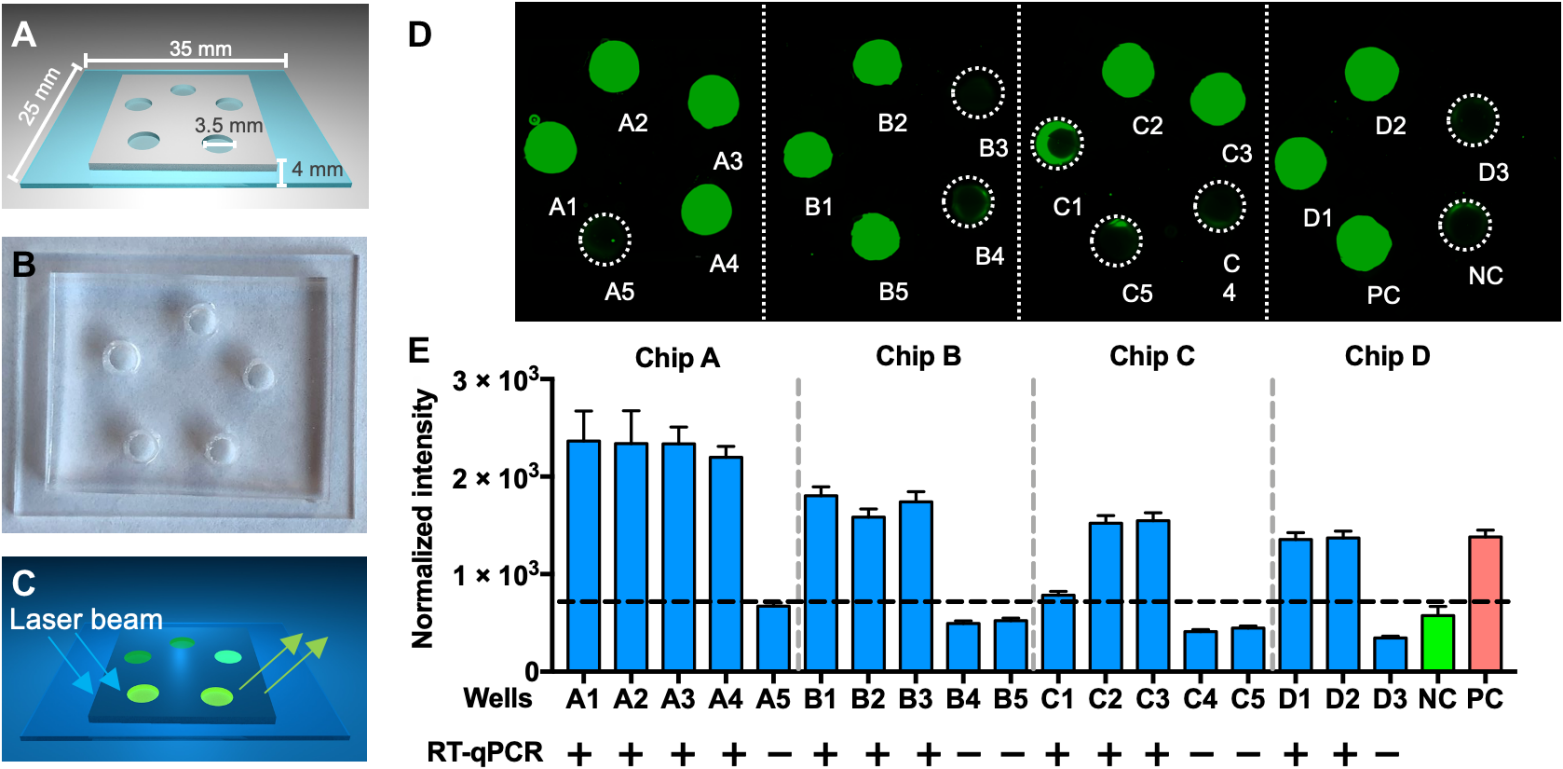
CRISPR-FDS chip prototype design and on-chip assay results. (**A**) Schematic of the on-chip design, (**B**) a prototype chip example, and (**C**) the assay readout schematic. (**D** and **E**) Fluorescence on-chip CRISPR-FDS assay images and signal for saliva from 11 patients diagnosed with COVID-19 and 7 patients diagnosed as non–COVID-19 cases by their nasal RT-qPCR results. (**D**) Fluorescence images were scanned and merged by a Cytation 5 Cell Imaging Multi-Mode Reader with 525-nm filter, and (**E**) assay fluorescence intensity was analyzed by the National Institutes of Health (NIH) ImageJ image analysis software. Data represent the means ± SD of three replicates. Photo Credit: Bo Ning, Tulane University.

Our chip design contained five reaction wells [internal diameter (I.D.) = 3.5 mm, maximum volume ≈ 28 μl] to allow the analysis of five assays in parallel (e.g., three test wells, one PC well, and one NC well), where wells were designed to contain sufficient volume for sensitive detection. Reaction wells were arranged in a pentagonal array illuminated by a laser diffused to cover the ~20 × 20 mm FOV of the smartphone camera (Fig. 4, A to C). A pentagonal array was chosen to minimize illumination differences in our proof-of-concept experiments; more compact arrays containing more wells could be used to simultaneously analyze samples from multiple individuals and/or to accommodate a standard curve to quantify viral load. Alternate designs could also use microfluidic channels to load multiple wells from a single inlet port and use film to seal the chip after sample loading to prevent environmental contamination by assay amplicons. To verify the use of this chip design, we used an on-chip CRISPR-FDS assay to analyze saliva from 12 patients with COVID-19 and 6 healthy controls. Images from this proof-of-concept assay approach that were captured and analyzed by a fluorescence microplate reader (Fig. 4, D and E) distinguished saliva from patients with positive and negative nasal RT-qPCR results, supporting the feasibility of this on-chip approach for COVID-19 diagnosis.

### Analytical and diagnostic performance of on-chip assays read by a smartphone device

**Fig. 5:**
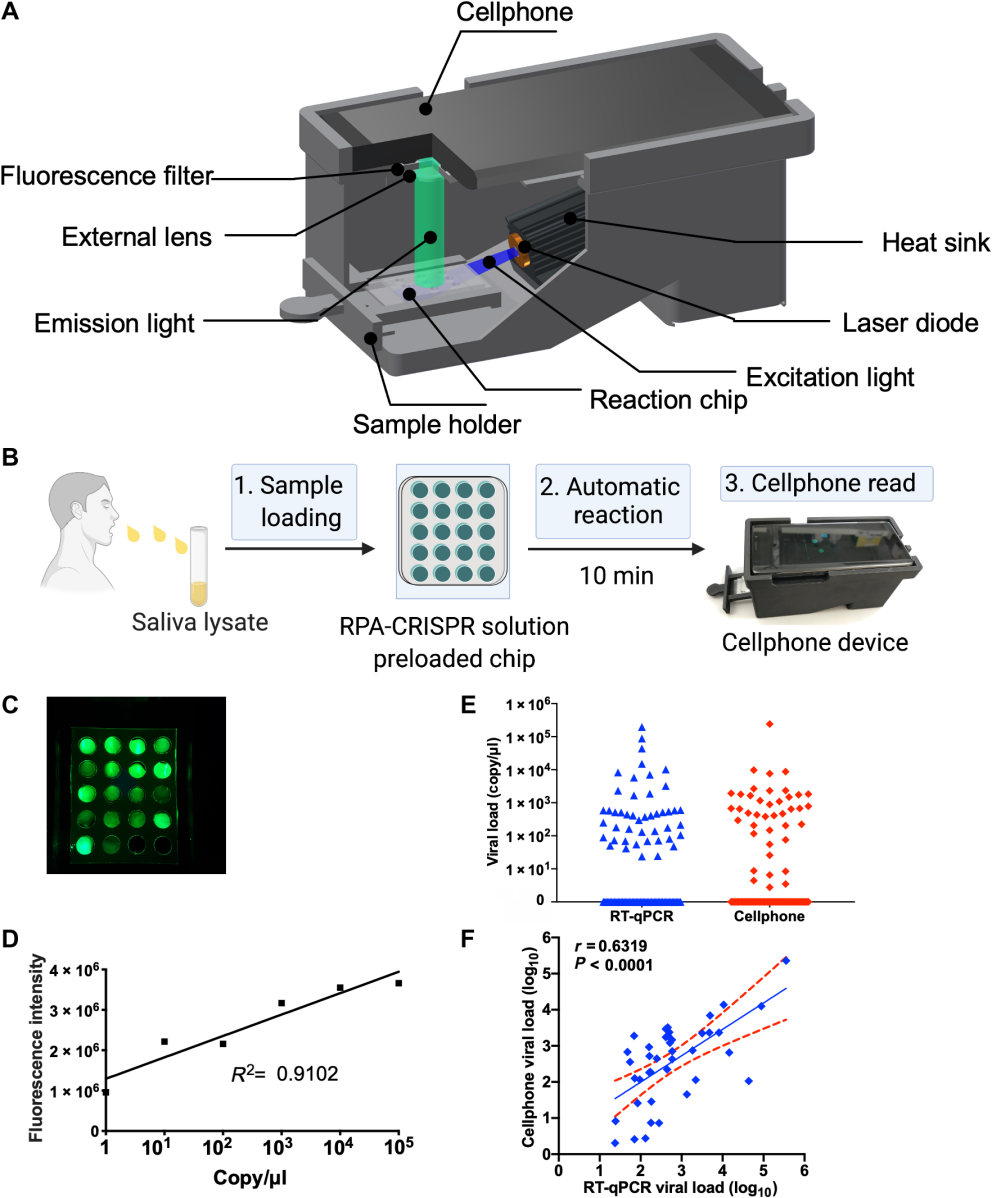
Design of a smartphone-based assay reader and its analytical and diagnostic performance when used to read on-chip CRISPR assays. (**A**) Schematic of a three-dimensional (3D)–printed smartphone fluorescence reader. (**B**) Workflow of a saliva-based on-chip CRISPR-FDS smartphone assay. (**C**) An example of CRISPR-FDS assay fluorescent signal images captured with a 525-nm filter with cellphone. (**D**) Standard curve of the on-chip CRISPR-FDS saliva test read by the smartphone device. (**E**) Comparison of SARS-CoV-2 viral load in saliva samples read by the smartphone device and by RT-PCR. Fluorescence intensity in smartphone images was analyzed by NIH ImageJ image analysis software. (**F**) Correlation of smartphone CRISPR-FDS and RT-qPCR assay results for saliva samples from 103 COVID-19 cases, indicating the linear regression line (solid) and the limits of its 95% CIs (dashed). Data represent the means ± SD of three replicates. Photo Credit: Bo Ning, Tulane University.

We next developed a smartphone fluorescence microscope device to read the on-chip CRISPR-FDS assay. This device incorporated a smartphone socket, external lens filter, laser diode powered by AAA batteries, a power switch, chip slot, and an emission filter for the smartphone camera (Fig. 5A). This integrated system was designed to use a straightforward workflow, in which a typical saliva volume of 0.5 to 3 ml (*35*) is collected in a tube prefilled with 3 ml of lysis buffer, which is then capped and heated at >37°C for ≥5 min, after which ~5 μl of the lysed sample is added to each sample well of an assay chip containing 10 μl per well of premixed RPA and CRISPR solution. This chip is then incubated for ≥10 min at room temperature and then inserted into the smartphone reader, the laser diode is turned on, and assay chip images are captured by the smartphone camera (Fig. 5B).

The FOV of this device was increased relative to previous smartphone microscope designs (*36*) by adding an external lens with a 50-mm focal length. This yielded an FOV compatible with the diameter of the reaction well array on our assay chip without producing notable aberration. This device also used a 100-mW laser diode with a high-incidence angle to allow sensitive detection of reaction products while minimizing background noise (Fig. 5C and fig. S3).

We examined the analytical performance of this device by analyzing on-chip assays of a SARS-CoV-2 RNA concentration curve generated serially diluting heat-inactivated SARS-CoV-2 virus in healthy donor saliva. This standard curve demonstrated good linearity (*R*^2^ = 0.91) over a broad viral concentration range (1 to 10^5^ copies/μl) and a calculated LOD of 0.38 copy/μl when read on the smartphone device (Fig. 5D) (*24*). We then used both CRISPR-FDS and RT-qPCR to blindly analyze 103 saliva samples from individuals screened for COVID-19 (Fig. 5E) and found that the CRISPR-FDS plate reader and smartphone assays and the standard RT-qPCR assay detected similar numbers of SARS-CoV-2–positive saliva samples (43 versus 44) (table S1). In an analysis using RT-qPCR as the reference standard (table S1), CRISPR smartphone results exhibited a 1.3% false-positive rate with saliva but complete concordance with RT-qPCR results for swab samples, while CRISPR plate reader results perfectly matched RT-qPCR saliva results, but exhibited a 2.3% false-negative rate with nasal swab samples. Viral load was strongly correlated in the 43 saliva samples that tested positive by both the on-chip smartphone assay and conventional RT-PCR analysis (Fig. 5F) and exhibited similar mean values (3803 versus 1797 copies/μl).

## DISCUSSION

New COVID-19 tests should ideally address several unmet needs. Rapid and ultrasensitive assays are needed to increase testing capacity and ideally should not require substantial equipment or technical expertise, or use diagnostic samples that must be collected by medical professionals. Sensitive and quantitative testing capacity is also needed to evaluate viral endpoint measures in a multitude of ongoing or planned clinical and animal studies for COVID-19–directed therapeutics or vaccines.

Recent studies have suggested that saliva may represent an ideal alternative diagnostic specimen for such assays. SARS-CoV-2 RNA was successfully detected in saliva samples collected from the deep throat region, oral cavity, and salivary gland of patients with COVID-19 (*25*–*27*), with viral load reported to be highest early after symptom onset (*37*). Moreover, saliva and nasopharyngeal samples are reported to exhibit very high PPA (96.6%) when samples were collected within 7 days of symptom onset, after which saliva appears to remain positive longer (*28*).

Virus loads during early infection cannot be accurately determined in patients with COVID-19 at specific time points postinfection because of uncertainty regarding initial infection events. However, our nonhuman primate data indicate that the SARS-CoV-2 viral load was much higher in oropharyngeal versus nasal swab samples at all time points when these samples were analyzed by CRISPR-FDS assay. SARS-CoV-2 RNA levels in analogous samples from rhesus macaques infected with a high intratracheal dose [10^6^ × TCID_50_ (median tissue culture infectious dose)] peaked at 3 days after infection and declined to undetectable levels within 10 days after infection (*38*); however, these animals developed mild to moderate disease and, thus, do not mimic the animals in our study, which developed asymptomatic infections. We also observed that saliva samples were more frequently SARS-CoV-2 positive than nasal swab samples when paired samples were tested by CRISPR-FDS and that saliva samples had higher mean viral loads when both samples were positive. Together, these results suggest that saliva may be a more sensitive diagnostic sample than nasal swabs.

Saliva levels of SARS-CoV-2 may also represent an important surrogate of virus infectivity, as viable virus can be easily cultured from saliva and saliva droplets are an important mechanism for virus transmission (*25*, *39*). Monitoring viral shedding in saliva should thus be of substantial interest as a potentially more direct and accurate means to evaluate infectivity in clinical practice and research studies.

Our results demonstrate that our saliva-based on-chip CRISPR-FDS assay for COVID-19 exhibits complete concordance with RT-qPCR when analyzing saliva samples spiked with SARS-CoV-2 concentrations that fall within the linear range of the RT-PCR assay, an estimated 0.38 copies/μl LOD, and a broad linear range (1 to 10^5^ copies/μl). Notably, this on-chip assay does not require RNA isolation but exhibits an LOD similar to RT-qPCR (0.38 copies/μl versus 1 copy/μl) and greater than CRISPR-based COVID-19 assays proposed for point-of-care diagnosis (4 to 10 copies/μl), all of which require separate RNA isolation procedures (*14*, *21*–*23*).

This assay platform has several features that should render it suitable for use in a variety of point-of-care testing environments, since it analyzes saliva samples that can be collected by the subject being tested to reduce the demands on medical personnel, exhibits robust performance in response to large variations in sample dilution and denaturation and CRISPR-FDS reaction temperatures and times, and uses an inexpensive and highly portable smartphone-based reader, which could also speed and simplify coded data reporting from remote testing sites.

Notably, the estimated sensitivity of our prototype smartphone device for SARS-CoV-2 in on-chip assays approaches the sensitivity we detect when off-chip assays are read by a fluorescence microplate reader (0.38 copies/μl versus 0.05 copies/μl), supporting the potential for broad use of this platform in screening and diagnosis. This sensitivity was achieved by low-incident angle illumination of the assay chip by a 100-mW laser diode powered by AAA batteries that achieved high excitation intensity and signal-to-noise conditions for assay well image capture. Sample focusing and image acquisition were achieved by the built-in smartphone camera app, eliminating the need for mechanical focusing and thus reducing weight and cost while enhancing the optical stability and user-friendliness of the device.

This proof-of-concept assay platform demonstrates the basic functionality required for a rapid ultrasensitive assay suitable for use in resource-limited point-of-care settings or screening sites. However, there are several refinements that could be made to further improve the user-friendliness and resource requirements of the current device. For example, our current protocol requires that saliva be lysed and then added to the chip for analysis, as do RT-RPA CRISPR-FDS reaction mixtures and controls, contributing additional sources of potential variation and error. We are therefore investigating the potential to control sample lysis and subsequent RT-PCR and CRISPR-FDS reactions on a microfluidic chip. In our anticipated next-generation chip and device design, saliva would be directly applied to a sample loading site on an integrated chip that would contain preloaded reagents and sample controls. Microfluidic channels on this chip would regulate the flow and mixing of reaction samples, while heating elements controlled by the smartphone would precisely regulate reaction temperatures. These chips could also be barcoded to facilitate data reporting. Last, a custom smartphone app would regulate chip temperature zones for the lysis, RT-RPA, and CRISPR-FDS reactions, automatically capture assay well images using the smartphone camera and analyze these data, and permit secure wireless reporting of assay data from remote sites to central locations to support telehealth efforts and provide aggregate data to governmental organizations tasked with making public health decisions.

We believe that this smartphone platform, or a similar future application, offers the potential to rapidly expand COVID-19 screening capacity and potentially simplify the verification of contact tracing, to improve local containment and inform regional disease control efforts.

## MATERIALS AND METHODS

### Key materials

The SuperScript IV One-Step RT-PCR System (1235820) and nuclease-free water (4387936) were purchased from Thermo Fisher Scientific (Waltham, MA). The EnGen Lba Cas12a (M0653T) and NEBuffer 2.1 (B7202S) were purchased from New England Biolabs (Ipswich, USA). Primers, gRNA, and probes (table S2) used in the study were synthesized by Integrated DNA Technologies Inc. (Coralville, IA). PDMS elastomer (Sylgard 184) was purchased from Dow Corning (Midland, MI), microscope glass slides were obtained from Thermo Fisher Scientific (Hampton, NH), and plasmonic oxidation was performed with a benchtop plasma cleaner PDC-001 purchased from Harrick Plasma (Ithaca, NY).

### Nucleic acid extraction

For assays analyzing isolated saliva RNA, total RNA was isolated from 100 μl of each saliva sample using a Quick DNA/RNA viral kit (Zymo; Hilden, Germany) and stored at −80°C until analysis.

### Quantitative reverse transcription polymerase chain reaction

RT-qPCR was performed with the CDC 2019-Novel Coronavirus (2019-nCoV) Real-Time RT-PCR Diagnostic Panel. In each reaction, 5 μl of RNA sample was mixed with 1 μl of Combined Primer/Probe Mix, 5 μl of TaqPath 4X 1-Step RT-qPCR Master Mix, and 9 μl of nuclease-free water. RT-qPCRs were performed using a QuantStudio 6 Flex Real-Time PCR System (Thermo Fisher Scientific, Waltham, USA) using the reaction conditions specified for this assay.

### RT-RPA amplification

RPA pellets from the TwistAmp Basic kit (ABAS03KIT; TwistDx Limited; Maidenhead, UK) were resuspended in 29.5 μl of the supplied rehydration buffer, and 11.8 μl of this RPA solution, 0.5 μl of forward primer (10 μM), 0.5 μl of reverse primer (10 μM), 3.2 μl of nuclease-free water, 4 μl of magnesium acetate (MgOAc; 280 mM), 1 μl SuperScript IV reverse transcriptase, and 5 of 60 μl isolated RNA sample were mixed and incubated at 42°C for 20 min.

### CRISPR-FDS reactions

For microplate-based CRISPR-FDS assays, 20 μl of a completed RT-RPA reaction was transferred to a 96-well half-area plate and mixed with 10 μl of a CRISPR reaction mixture containing 3 μl of 10× NEBuffer 2.1, 3 μl of gRNA (300 nM), 1 μl of EnGen Lba Cas12a (1 μM), 1.5 μl of fluorescent probe (10 μM), and 1.5 μl of nuclease-free water. After incubation at 37°C, for 20 min in the dark, fluorescence signal was detected using SpectraMax i3x Multi-Mode Microplate Reader (Molecular Devices LLC, San Jose, USA). For the Cas12a substrate–dependent kinetics study, the CRISPR-FDA reaction contained 1:15, 1:20, and 1:25 molar ratios of Cas12a/gRNA to fluorescent probe. For the temperature-dependent kinetics study, reactions were performed using a 1:25 Cas12a/gRNA–to–fluorescent probe ratio at 22°, 37°, and 42°C.

### RNA extraction–free saliva assay

QuickExtract DNA Extraction Solution (Lucigen) was mixed with saliva samples as indicated to release viral RNA, since this solution is compatible with PCR, RPA, and CRISPR reactions (*40*). Saliva and lysis buffer mixtures were then incubated at the temperatures and times specified, after which 5 μl of the lysed sample was mixed with an RT-RPA solution described above and incubated at 37°C to amplify a SARS-CoV-2 ORF1ab gene target, which was detected by the addition of a 10-μl mixture containing a CRISPR-FDS reaction mixture described above. For the on-chip CRISPR-FDS assays, 10 μl of an RT-RPA CRISPR solution generated by mixing equal volumes of the RT-RPA and CRISPR-FDS reaction mixtures was preloaded into each chip well, after which 5 μl of saliva lysate or purified RNA was loaded into these wells, and the chip was incubated at ≥22°C for ≥5 min.

### Standard curve, LOD, and LOD detection rates

Standard curve was generated by spiking a known amount of heat-inactivated virus into healthy donor saliva and then serially diluting this starting sample with healthy human saliva, as indicated for the respective assay. LOD was calculated using the mean and SD of the zero control (blank) and lowest concentration standard following the formula: LOD = mean_blank_ + 1.645_(SDblank)_ + 1.645_(SD low concentration sample)_ (*24*). To assess detection rates near the estimated LOD, healthy donor saliva was spiked with heat-inactivated SARS-CoV-2 virus at 0.25 copy/μl and serially diluted to 0.1 and 0.05 copy/μl concentrations. RNA was extracted from each of these dilution samples by three different individuals to generate three RNA batches. For RT-RPA CRISPR-FDS assays, each extracted RNA batch was analyzed in 20 replicates for a total of 60 replicates among the three batches generated at each concentration. A positive sample was defined as any sample with a CRISPR-FDS signal that was greater than the mean signal of the negative control plus three times its SD.

### Cross-reactivity (analytical specificity)

#### In silico testing

A total of 39 bacterial/viral/fungal strains have been analyzed in silico. NCBI BLAST tool was used to check for cross-reactivity of the primer/gRNA sets of the SARS-CoV-2 assay against the nonredundant nucleotide database. The default parameters of BLAST tool were used except for the “organism.” The search was limited to using the taxonomy ID (taxid/txid) of the respective pathogen. Each primer and gRNA were compared against all the available genomic sequences of a certain taxid.

#### Wet testing

To confirm the in silico data, we conducted the wet testing of the high-risk pathogenic microorganisms commonly seen in the respiratory tract. Each microorganism listed in table S3 was analyzed in triplicate with our SARS-CoV-2 CRISPR by spiking diluted organism stock into lysis-treated pooled nasopharyngeal swab matrix.

### Nonhuman primate model

#### Ethics statement

The Institutional Animal Care and Use Committee of Tulane University reviewed and approved all the procedures for this experiment. The Tulane National Primate Research Center is fully accredited by the Association for Assessment and Accreditation of Laboratory Animal Care (AAALAC). All animals are cared for in accordance with the National Institutes of Health (NIH) Guide for the Care and Use of Laboratory Animals. The Tulane Institutional Biosafety Committee approved the procedures for sample handling, inactivation, and removal from BSL3 containment.

#### Virus information

SARS-CoV-2 isolate USA-WA1/2020 was acquired from BEI Resources, and the harvested stock was determined to have a concentration of 1 × 10^6^ TCID_50_/ml. The virus was passaged in Vero E6 cells in DMEM (Dulbecco’s modified Eagle’s medium) media with 2% fetal bovine serum sequence confirmed by PCR and/or Sanger sequencing. Plaque assays were performed in Vero E6 cells.

#### Animals and procedures

A total of seven nonhuman primates aged >11 years (two male and one female African green monkey and two male and two female Indian rhesus macaques) were analyzed in this study. Animals were exposed to SARS-CoV-2 either by multiroute combination or small-particle aerosolization. Animals exposed by aerosol received an inhaled dose of ~2.5 × 10^4^ plaque-forming units (PFU). Animals exposed by multiroute combination (oral, 1 ml; nasal, 1 ml; intratracheal, 1 ml; conjunctiva, 50 μl per eye) received a cumulative dose of 3.61 × 10^6^ PFU. Animals were observed for 28 days after infection including twice daily monitoring by veterinary staff.

### Design of the smartphone device for quantification of the on-chip CFRISPR-FDS assay

This device is composed of two components: a Samsung Galaxy S9 smartphone with a 12.2-megapixel (5312 × 2988) 7.06-mm CMOS image sensor and a rear camera with a 26-mm focal length, and a custom printed fluorescence microscope interface that was designed in Autodesk Inventor, prepared by three-dimensional (3D) printing (StrataSys uPrint SE plus) and then fitted with optomechanical attachments. A blue laser diode (465 nm, >100 mW, DTR’s Laser Shop) powered by three AAA batteries was connected to a heatsink and mounted to the 3D-printed base, as was a convex lens (*f*_2_ = 50 mm, Edmund Optics, #38-296) for signal collection with a demagnification index calculated as *M* = *f*_1_/*f*_2_ ≈ 0.52, and a 525-nm band-pass filter (Edmund Optics, #86-354) that was placed in front of the smartphone camera as an emission. This device was also fitted with a sample tray to accurately position the assay chip during its readout.

### Capture and analysis of smartphone CRISPR-FDS reaction well images

Assay chips containing completed CRISPR-FDS reactions were inserted into the smartphone reader, and images of chip fluorescent signal were captured using the Samsung Galaxy S9 smartphone camera app Version 9.0.05 using the manual focusing function in this app. All samples analyzed on the Samsung smartphone device were imaged using 1/15 s integration time and an ISO value of 100, and captured images were stored using a lossless raw format (DNG file). Raw smartphone DNG images were first transferred to a computer through a USB cable and converted to RGB TIFF images via DC RAW V 1.5.0. A monochromic TIFF image was generated by extracting the green (G) channel values for further analysis, where pixel intensity from this monochromic image was then measured and analyzed using ImageJ.

### Clinical samples

Nasal swab and saliva specimens were collected at Tulane Medical Center in New Orleans, LA, from 30 March to 16 July 2020 with written informed consent, in accordance with an approved institutional review board (IRB) protocol, and the COVID-19 status of the donors was determined on the basis of clinical indications and current CDC guidance; all nasal swab samples were tested with the CDC 2019-nCoV Real-Time RT-PCR Diagnostic Panel (EUA). Saliva from a patient with Philadelphia-negative B cell acute lymphoblastic leukemia analyzed in this study, and previously reported in a case study (*29*), was provided by N. Saba under a separate IRB. Clinical specimens from COVID-19–infected subjects were processed in an enhanced BL2/BL3 space at Tulane University in accordance with a protocol approved by the Institutional Biosafety Committee.

### Statistical analysis

CRISPR-FDS assay signal was expressed as the mean of ≥3 independent reactions ± SD. GraphPad Prism 8 was used to calculate one-way analysis of variance (ANOVA), determine the optimized condition of RT-RPA, and calculate linear regression of standard curve. Multiple-group comparisons were conducted using one-way ANOVA. Differences were considered statistically significance at *P* < 0.05.

## Supplementary Material

http://advances.sciencemag.org/cgi/content/full/sciadv.abe3703/DC1

Adobe PDF - abe3703_SM.pdf

A smartphone-read ultrasensitive and quantitative saliva test for COVID-19

## SUPPLEMENTARY MATERIALS

Supplementary material for this article is available at http://advances.sciencemag.org/cgi/content/full/sciadv.abe3703/DC1

## References

[1] Zhou P., Yang X.-L., Wang X.-G., Hu B., Zhang L., Zhang W., Si H.-R., Zhu Y., Li B., Huang C.-L., Chen H.-D., Chen J., Luo Y., Guo H., Jiang R.-D., Liu M.-Q., Chen Y., Shen X.-R., Wang X., Zheng X.-S., Zhao K., Chen Q.-J., Deng F., Liu L.-L., Yan B., Zhan F.-X., Wang Y.-Y., Xiao G.-F., Shi Z.-L., A pneumonia outbreak associated with a new coronavirus of probable bat origin. Nature 579, 270–273 (2020). 32015507 10.1038/s41586-020-2012-7 PMC7095418

[2] Liu Q., Liu Z., Zhu J., Zhu Y., Li D., Gao Z., Zhou L., Tang Y., Zhang X., Yang J., Wang Q., Assessing the global tendency of COVID-19 outbreak. medRxiv 2020.03.18.20038224 (2020).

[3] Péré H., Podglajen I., Wack M., Flamarion E., Mirault T., Goudot G., Hauw-Berlemont C., Le L., Caudron E., Carrabin S., Rodary J., Ribeyre T., Bélec L., Veyer D., Nasal swab sampling for SARS-CoV-2: A convenient alternative in times of nasopharyngeal swab shortage. J. Clin. Microbiol. 58, e00721-20 (2020). 32295896 10.1128/JCM.00721-20 PMC7269411

[4] Spencer S., Thompson M. G., Flannery B., Fry A., Comparison of respiratory specimen collection methods for detection of influenza virus infection by reverse transcription-PCR: A literature review. J. Clin. Microbiol. 57, e00027-19 (2019). 31217267 10.1128/JCM.00027-19 PMC6711916

[5] Udugama B., Kadhiresan P., Kozlowski H. N., Malekjahani A., Osborne M., Li V. Y. C., Chen H., Mubareka S., Gubbay J. B., Chan W. C. W., Diagnosing COVID-19: The disease and tools for detection. ACS Nano 14, 3822–3835 (2020). 32223179 10.1021/acsnano.0c02624

[6] CDC 2019-Novel Coronavirus (2019-nCoV) Real-Time RT-PCR Diagnostic Panel (2020). 10.1371/journal.pone.0260487 PMC8673615 34910739

[7] Smithgall M. C., Scherberkova I., Whittier S., Green D. A., Comparison of Cepheid Xpert Xpress and Abbott ID Now to Roche cobas for the rapid detection of SARS-CoV-2. J. CLIN. VIROL. 128, 104428 (2020). 32434706 10.1016/j.jcv.2020.104428 PMC7217789

[8] Harrington A., Cox B., Snowdon J., Bakst J., Ley E., Grajales P., Maggiore J., Kahn S., Comparison of Abbott ID Now and Abbott m2000 methods for the detection of SARS-CoV-2 from nasopharyngeal and nasal swabs from symptomatic patients. J. Clin. Microbiol. 58, e00798-20 (2020). 32327448 10.1128/JCM.00798-20 PMC7383519

[9] Hogan C. A., Sahoo M. K., Huang C. H., Garamani N., Stevens B., Zehnder J., Pinsky B. A., Five-minute point-of-care testing for SARS-CoV-2: Not there yet. J. Clin. Virol. 128, 104410 (2020). 32403009 10.1016/j.jcv.2020.104410 PMC7194071

[10] Gootenberg J. S., Abudayyeh O. O., Lee J. W., Essletzbichler P., Dy A. J., Joung J., Verdine V., Donghia N., Daringer N. M., Freije C. A., Myhrvold C., Bhattacharyya R. P., Livny J., Regev A., Koonin E. V., Hung D. T., Sabeti P. C., Collins J. J., Zhang F., Nucleic acid detection with CRISPR-Cas13a/C2c2. Science 356, 438–442 (2017). 28408723 10.1126/science.aam9321 PMC5526198

[11] Chen J. S., Ma E., Harrington L. B., Costa M. D., Tian X., Palefsky J. M., Doudna J. A., CRISPR-Cas12a target binding unleashes indiscriminate single-stranded DNase activity. Science 360, 436–439 (2018). 29449511 10.1126/science.aar6245 PMC6628903

[12] Li S.-Y., Cheng Q.-X., Wang J.-M., Li X.-Y., Zhang Z.-L., Gao S., Cao R.-B., Zhao G.-P., Wang J., CRISPR-Cas12a-assisted nucleic acid detection. Cell Discov 4, 20 (2018). 29707234 10.1038/s41421-018-0028-z PMC5913299

[13] Pardee K., Green A. A., Takahashi M. K., Braff D., Lambert G., Lee J. W., Ferrante T., Ma D., Donghia N., Fan M., Daringer N. M., Bosch I., Dudley D. M., O’Connor D. H., Gehrke L., Collins J. J., Rapid, low-cost detection of zika virus using programmable biomolecular components. Cell 165, 1255–1266 (2016). 27160350 10.1016/j.cell.2016.04.059

[14] Lucia C., Federico P.-B., Alejandra G. C., An ultrasensitive, rapid, and portable coronavirus SARS-CoV-2 sequence detection method based on CRISPR-Cas12. bioRxiv 2020.02.29.971127 (2020).

[15] Bruch R., Baaske J., Chatelle C., Meirich M., Madlener S., Weber W., Dincer C., Urban G. A., CRISPR/Cas13a-powered electrochemical microfluidic biosensor for nucleic acid amplification-free miRNA diagnostics. Adv. Mater. 31, 1905311 (2019). 10.1002/adma.201905311 31663165

[16] Bruch R., Urban G. A., Dincer C., CRISPR/Cas powered multiplexed biosensing. Trends Biotechnol. 37, 791–792 (2019). 31078316 10.1016/j.tibtech.2019.04.005

[17] Hajian R., Balderston S., Tran T., deBoer T., Etienne J., Sandhu M., Wauford N. A., Chung J.-Y., Nokes J., Athaiya M., Paredes J., Peytavi R., Goldsmith B., Murthy N., Conboy I. M., Aran K., Detection of unamplified target genes via CRISPR–Cas9 immobilized on a graphene field-effect transistor. Nat. Biomed. Eng. 3, 427–437 (2019). 31097816 10.1038/s41551-019-0371-x PMC6556128

[18] Li Y., Li S., Wang J., Liu G., CRISPR/Cas systems towards next-generation biosensing. Trends Biotechnol. 37, 730–743 (2019). 30654914 10.1016/j.tibtech.2018.12.005

[19] Zhang Y., Odiwuor N., Xiong J., Sun L., Nyaruaba R. O., Wei H., Tanner N. A., Rapid molecular detection of SARS-CoV-2 (COVID-19) virus RNA using colorimetric LAMP. medRxiv 2020.02.26.20028373 (2020).

[20] Zaghloul H., El-Shahat M., Recombinase polymerase amplification as a promising tool in hepatitis C virus diagnosis. World J. Hepatol. 6, 916–922 (2014). 25544878 10.4254/wjh.v6.i12.916 PMC4269910

[21] Ding X., Yin K., Li Z., Liu C., All-in-One Dual CRISPR-Cas12a (AIOD-CRISPR) Assay: A case for rapid, ultrasensitive and visual detection of novel coronavirus SARS-CoV-2 and HIV virus. bioRxiv 2020.03.19.998724 (2020). 10.1038/s41467-020-18575-6 PMC7501862 32948757

[22] Yu L., Wu S., Hao X., Li X., Liu X., Ye S., Han H., Dong X., Li X., Li J., Liu N., Liu J., Zhang W., Pelechano V., Chen W.-H., Yin X., Rapid colorimetric detection of COVID-19 coronavirus using a reverse tran-scriptional loop-mediated isothermal amplification (RT-LAMP) diagnostic plat-form: iLACO. medRxiv 2020.02.20.20025874 (2020). 10.1093/clinchem/hvaa102 PMC7188121 32315390

[23] Broughton J. P., Deng X., Yu G., Fasching C. L., Servellita V., Singh J., Miao X., Streithorst J. A., Granados A., Sotomayor-Gonzalez A., Zorn K., Gopez A., Hsu E., Gu W., Miller S., Pan C.-Y., Guevara H., Wadford D. A., Chen J. S., Chiu C. Y., CRISPR–Cas12-based detection of SARS-CoV-2. Nat. Biotechnol. 38, 870–874 (2020). 32300245 10.1038/s41587-020-0513-4 PMC9107629

[24] Armbruster D. A., Pry T., Limit of blank, limit of detection and limit of quantitation. Clin. Biochem. Rev. 29 (suppl. 1), S49–S52 (2008). 18852857 PMC2556583

[25] To K. K.-W., Tsang O. T.-Y., Yip C. C.-Y., Chan K.-H., Wu T.-C., Chan J. M.-C., Leung W.-S., Chik T. S.-H., Choi C. Y.-C., Kandamby D. H., Lung D. C., Tam A. R., Poon R. W.-S., Fung A. Y.-F., Hung I. F.-N., Cheng V. C.-C., Chan J. F.-W., Yuen K.-Y., Consistent detection of 2019 novel coronavirus in saliva. Clin. Infect. Dis. 71, 841–843 (2020). 32047895 10.1093/cid/ciaa149 PMC7108139

[26] Zhang W., Du R.-H., Li B., Zheng X.-S., Yang X.-L., Hu B., Wang Y.-Y., Xiao G.-F., Yan B., Shi Z.-L., Zhou P., Molecular and serological investigation of 2019-nCoV infected patients: Implication of multiple shedding routes. Emerg. Microbes Infect. 9, 386–389 (2020). 32065057 10.1080/22221751.2020.1729071 PMC7048229

[27] L. Chen, J. Zhao, J. Peng, X. Li, X. Deng, Z. Geng, Z. Shen, F. Guo, Q. Zhang, Y. Jin, L. Wang, S. Wang, Detection of 2019-nCoV in saliva and characterization of oral symptoms in COVID-19 patients. Available at SSRN 3556665 (2020). 10.1111/cpr.12923 PMC7645955 33073910

[28] Cheuk S., Wong Y., Tse H., Siu H. K., Kwong T. S., Chu M. Y., Yau F. Y. S., Cheung I. Y. Y., Tse C. W. S., Poon K. C., Cheung K. C., Wu T. C., Chan J. W. M., Cheuk W., Posterior oropharyngeal saliva for the detection of SARS-CoV-2. Clin. Infect. Dis., ciaa797 (2020). 10.1093/cid/ciaa797 PMC7337706 32562544

[29] Niu A., McDougal A., Ning B., Safa F., Luk A., Mushatt D. M., Nachabe A., Zwezdaryk K. J., Robinson J., Peterson T., Socola F., Safah H., Hu T., Saba N. S., COVID-19 in allogeneic stem cell transplant: High false-negative probability and role of CRISPR and convalescent plasma. Bone Marrow Transplant 55, 2354–2356 (2020). 32541689 10.1038/s41409-020-0972-8 PMC7294520

[30] Zhang S., Li Z., Wei Q., Smartphone-based cytometric biosensors for point-of-care cellular diagnostics. Nanotechnol. Precision Eng. 3, 32–42 (2020).

[31] Hernández-Neuta I., Neumann F., Brightmeyer J., Tis T. B., Madaboosi N., Wei Q., Ozcan A., Nilsson M., Smartphone-based clinical diagnostics: Towards democratization of evidence-based health care. J. Intern. Med. 285, 19–39 (2019). 30079527 10.1111/joim.12820 PMC6334517

[32] Joh D. Y., Hucknall A. M., Wei Q., Mason K. A., Lund M. L., Fontes C. M., Hill R. T., Blair R., Zimmers Z., Achar R. K., Tseng D., Gordan R., Freemark M., Ozcan A., Chilkoti A., Inkjet-printed point-of-care immunoassay on a nanoscale polymer brush enables subpicomolar detection of analytes in blood. Proc. Natl. Acad. Sci. U.S.A. 114, E7054–E7062 (2017). 28784765 10.1073/pnas.1703200114 PMC5576789

[33] Kong J. E., Wei Q., Tseng D., Zhang J., Pan E., Lewinski M., Garner O. B., Ozcan A., Carlo D. D., Highly stable and sensitive nucleic acid amplification and cell-phone-based readout. ACS Nano 11, 2934–2943 (2017). 28234452 10.1021/acsnano.6b08274

[34] Kühnemund M., Wei Q., Darai E., Wang Y., Hernández-Neuta I., Yang Z., Tseng D., Ahlford A., Mathot L., Sjöblom T., Ozcan A., Nilsson M., Targeted DNA sequencing and *in situ* mutation analysis using mobile phone microscopy. Nat. Commun. 8, 13913 (2017). 28094784 10.1038/ncomms13913 PMC5247573

[35] Pulford D. J., Mosteller M., Briley J. D., Johansson K. W., Nelsen A. J., Saliva sampling in global clinical studies: The impact of low sampling volume on performance of DNA in downstream genotyping experiments. BMC Med. Genomics 6, 20 (2013). 23759220 10.1186/1755-8794-6-20 PMC3698156

[36] Vietz C., Schütte M. L., Wei Q., Richter L., Lalkens B., Ozcan A., Tinnefeld P., Acuna G. P., Benchmarking smartphone fluorescence-based microscopy with DNA origami nanobeads: Reducing the gap toward single-molecule sensitivity. ACS Omega 4, 637–642 (2019). 30775643 10.1021/acsomega.8b03136 PMC6372172

[37] To K. K.-W., Tsang O. T.-Y., Leung W.-S., Tam A. R., Wu T.-C., Lung D. C., Yip C. C.-Y., Cai J.-P., Chan J. M.-C., Chik T. S.-H., Lau D. P.-L., Choi C. Y.-C., Chen L.-L., Chan W.-M., Chan K.-H., Ip J. D., Ng A. C.-K., Poon R. W.-S., Luo C.-T., Cheng V. C.-C., Chan J. F.-W., Hung I. F.-N., Chen Z., Chen H., Yuen K.-Y., Temporal profiles of viral load in posterior oropharyngeal saliva samples and serum antibody responses during infection by SARS-CoV-2: An observational cohort study. Lancet Infect. Dis. 20, 565–574 (2020). 32213337 10.1016/S1473-3099(20)30196-1 PMC7158907

[38] Deng W., Bao L., Liu J., Xiao C., Liu J., Xue J., Lv Q., Qi F., Gao H., Yu P., Xu Y., Qu Y., Li F., Xiang Z., Yu H., Gong S., Liu M., Wang G., Wang S., Song Z., Liu Y., Zhao W., Han Y., Zhao L., Liu X., Wei Q., Qin C., Primary exposure to SARS-CoV-2 protects against reinfection in rhesus macaques. Science 369, 818–823 (2020). 32616673 10.1126/science.abc5343 PMC7402625

[39] Stadnytskyi V., Bax C. E., Bax A., Anfinrud P., The airborne lifetime of small speech droplets and their potential importance in SARS-CoV-2 transmission. Proc. Natl. Acad. Sci. U.S.A. 117, 11875 (2020). 32404416 10.1073/pnas.2006874117 PMC7275719

[40] Zetsche B., Heidenreich M., Mohanraju P., Fedorova I., Kneppers J., De Gennaro E. M., Winblad N., Choudhury S. R., Abudayyeh O. O., Gootenberg J. S., Wu W. Y., Scott D. A., Severinov K., van der Oost J., Zhang F., Multiplex gene editing by CRISPR–Cpf1 using a single crRNA array. Nat. Biotechnol. 35, 31–34 (2017). 27918548 10.1038/nbt.3737 PMC5225075

