# Supplementary material for: A smartphone-read ultrasensitive and quantitative saliva test for COVID-19
Source: Sci Adv. 2021 Jan 8;7(2):eabe3703. doi: 10.1126/sciadv.abe3703 (PMC7793573; doi:10.1126/sciadv.abe3703)
Supplement: http://advances.sciencemag.org/cgi/content/full/sciadv.abe3703/DC1 [file abe3703_index.html]

Science Advances | Science AdvancesAAASSearchScience AdvancesMenu

## Supplementary Materials

# A smartphone-read ultrasensitive and quantitative saliva test for COVID-19

Bo Ning, Tao Yu, Shengwei Zhang, Zhen Huang, Di Tian, Zhen Lin, Alex Niu, Nadia Golden, Krystle Hensley, Breanna Threeton, Christopher J. Lyon, Xiao-Ming Yin, Chad J. Roy, Nakhle S. Saba, Jay Rappaport, Qingshan Wei and Tony Y. Hu

Download Supplement

**This PDF file includes:**

- Tables S1 to S3
- Figs. S1 to S3

**Files in this Data Supplement:**

- Adobe PDF - abe3703\_SM.pdf
