# Supplementary material for: A smartphone-read ultrasensitive and quantitative saliva test for COVID-19
Source: Sci Adv. 2021 Jan 8;7(2):eabe3703. doi: 10.1126/sciadv.abe3703 (PMC7793573; doi:10.1126/sciadv.abe3703)
Supplement: http://advances.sciencemag.org/cgi/content/full/sciadv.abe3703/DC1 [file abe3703_1.pdf]

Science Advances
AAAS

advances.sciencemag.org/cgi/content/full/sciadv.abe3703/DC1

Supplementary Materials for

A smartphone-read ultrasensitive and quantitative saliva test for COVID-19

Bo Ning, Tao Yu, Shengwei Zhang, Zhen Huang, Di Tian, Zhen Lin, Alex Niu, Nadia Golden, Krystle Hensley, Breanna Threeton, Christopher J. Lyon, Xiao-Ming Yin, Chad J. Roy, Nakhle S. Saba, Jay Rappaport, Qingshan Wei and Tony Y. Hu*

*Corresponding author. Email: tonyhu@tulane.edu

Published 11 December 2020, *Sci. Adv.* **6**, eabe3703 (2020)
DOI: 10.1126/sciadv.abe3703

This PDF file includes:

Tables S1 to S3
Figs. S1 to S3

Table S1: Summary of RT-qPCR, CRISPR smartphone and plate reader assay results.

		Positive	Negative
RT-qPCR	Saliva	44	59
	Nasal swab	27	76
Smartphone	Saliva	43	60
	Nasal swab	27	76
Plate reader	Saliva	44	59
	Nasal swab	28	75

Table S2: Oligonucleotide list

Name	Sequence	Target Gene
**For RT-PCR/CRISPR and RT-RPA/CRISPR**		
ORF1ab-F	CCCTGTGGGTTTTACACTTAA	ORF1ab
ORF1ab-R	ACGATTGTGCATCAGCTGA	ORF1ab
gRNA -ORF1ab	UAAUUUCUACUCUUGUAGAUCACAUACC GCAGACGGUACAGAC	ORF1ab
N Forward Primer	GGGGAACTTCTCCTGCTAGAAT	N-gene
N Reverse Primer	CAGACATTTTGCTCTCAAGCTG	N-gene
N gRNA	UAAUUUCUACUCUUGUAGAUCUGCUGCU UGACAGAUUGAAC	N-gene
RPP30 Forward Primer	CTCGGATCCATCTCACTGCAA	RPP30
RPP30 Reverse Primer	TGCAACAACATCATAGAGCCG	RPP30
RPP30 gRNA	UAAUUUCUACUCUUGUAGAUAGAGCAAC UUCUUCAAGGGCCC	RPP30
Fluorescent reporter	FAM-TTTTTTTTTTTT-BHQ	
**For RT-qPCR**		
USCDC-N2-F	TTACAAACATTGGCCGCAAA	N-gene
USCDC-N2-R	GCGCGACATTCCGAAGAA	N-gene
USCDC-N2-Probe	FAM-ACAATTTGCCCCCAGCGCTTCAG-BHP1	N-gene

Table S3. CRISPR-FDS specificity for common respiratory flora and other viral pathogens.

Microorganism	Supplier	Item No.	Sample Type	Concentration	ORF1ab	N	RPP30
Bordetella pertussis I176	BEI	NR-42463	Particles	>10^6 copies/ml	0/3	0/3	3/3
Candida albicans 23B	BEI	NR-19340	Particles	>10^6 copies/ml	0/3	0/3	3/3
Candida albicans	BEI	NR-50361	Genomic RNA	10 ng/100 ul	0/3	0/3	3/3
Chlamydia pneumoniae	ATCC	VR-1356	Particles	>10^5 copies/ml	0/3	0/3	3/3
Enterovirus 71	BEI	NR-4961	Genomic RNA	10 ng/100 ul	0/3	0/3	3/3
Haemophilus influenzae	ATCC	51907DQ	Genomic DNA	>10^5 copies/ml	0/3	0/3	3/3
Human parainfluenza Virus 2	BEI	NR-3229	Particles	>10^5 copies/ml	0/3	0/3	3/3
Human parainfluenza Virus 3	BEI	NR-3233	Particles	>10^5 copies/ml	0/3	0/3	3/3
Human adenovirus	ATCC	VR-1D	Genomic DNA	>10^5 copies/ml	0/3	0/3	3/3
Human coronavirus 229E	BEI	NR-57276	Particles	>10^5 copies/ml	0/3	0/3	3/3
Human coronavirus NL63	BEI	NR-470	Particles	>10^5 copies/ml	0/3	0/3	3/3
Human coronavirus OC43	BEI	NR-52725	Particles	>10^5 copies/ml	0/3	0/3	3/3
Human coronavirus HKU1	ATCC	VR-3262SD	Synthetic RNA	>10^5 copies/ml	0/3	0/3	3/3
Human coronavirus NL63	BEI	NR-470	Genomic RNA	10 ng/100 ul	0/3	0/3	3/3
Human metapneumovirus	BEI	NR-49122	Genomic RNA	10 ng/100 ul	0/3	0/3	3/3
Human parainfluenza	ATCC	VR-94DQ	Genomic DNA	>10^5 copies/ml	0/3	0/3	3/3
Human parainfluenza virus 4b	ATCC	VR-1377D	Genomic RNA	>10^5 copies/ml	0/3	0/3	3/3
Influenza A	BEI	NR-2760	Genomic RNA	10 ng/100 ul	0/3	0/3	3/3
Influenza A	BEI	NR-41800	Particles	>10^5 copies/ml	0/3	0/3	3/3
Influenza B	BEI	NR-10048	Genomic RNA	10 ng/100 ul	0/3	0/3	3/3

Legionella pneumophila	ATCC	33152DQ	Genomic DNA	>10^5 copies/ml	0/3	0/3	3/3
MERS-coronavirus	ATCC	VR-3248SD	Synthetic RNA	>10^5 copies/ml	0/3	0/3	3/3
Mycobacterium tuberculosis	BEI	NR-14867	Genomic DNA	10 ng/100 ul	0/3	0/3	3/3
Mycoplasma pneumoniae	ATCC	29342DQ	Genomic DNA	>10^5 copies/ml	0/3	0/3	3/3
Pneumocystis jirovecii (PJP)	ATCC	PRA-159	Particles	>10^5 copies/ml	0/3	0/3	3/3
Pseudomonas aeruginosa	BEI	NR-51329	Particles	>10^6 copies/ml	0/3	0/3	3/3
Respiratory syncytial virus	BEI	NR-43976	Genomic RNA	10 ng/100 ul	0/3	0/3	3/3
Rhinovirus 40	BEI	NR-51453	Particles	>10^6 copies/ml	0/3	0/3	3/3
SARS-coronavirus	ATCC	VR-3280SD	Synthetic RNA	>10^5 copies/ml	0/3	0/3	3/3
SARS-coronavirus	BEI	NR-9323	Particles	>10^5 copies/ml	0/3	0/3	3/3
Staphylococcus epidermis	BEI	NR-51354	Genomic DNA	10 ng/100 ul	0/3	0/3	3/3
Streptococcus salivarius	BEI	HM-121	Particles	>10^6 copies/ml	0/3	0/3	3/3
Streptococcus pneumoniae	BEI	NR-51849	Particles	>10^6 copies/ml	0/3	0/3	3/3
Streptococcus pyogenes	BEI	NR-48702	Particles	>10^6 copies/ml	0/3	0/3	3/3
Streptococcus salicarius	BEI	HM-121	Particles	>10^6 copies/ml	0/3	0/3	3/3

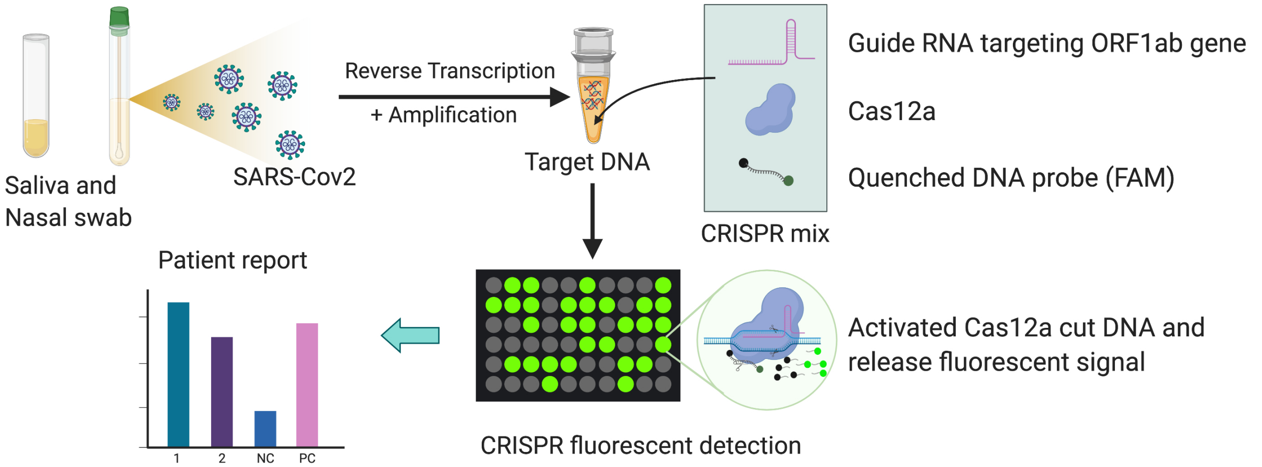

Figure S1. CRISPR-FDS assay workflow and mechanism for SARS-CoV-2 RNA detection. CRISPR-FDS utilizes integrates simultaneous isothermal reverse transcriptase and RPA reactions that amplify a target amplicon region that is then transiently bound in a sequence-specific manner by a CRISPR-Cas12a guide RNA (gRNA) complex, determined by the gRNA sequence (ORF1ab). Cas12a/gRNA binding activates this enzyme complex to rapidly and nonspecifically cleave an interacting single-stranded polyT DNA oligonucleotide probe present in large molar excess. Cleavage of the assay probe unmasks its quenched fluorescent label to produce fluorescent in proportion to amount of available amplicon in the reaction, which directly reflects the amount of SARS-CoV-2 RNA present in the analysis sample. CRISPR-FDS fluorescent signal development is rapid since probe cleavage occurs in parallel with the amplification of its sequence target, producing a fluorescent signal that can be sensitively read by benchtop plate reader or cellphone-based chip reader, and compared to negative and positive control (NC and PC) samples and concentration standards to detect and quantify the amount of SARS-CoV-2 RNA present in the analyzed sample. CRISPR-FDS assay primers, probes, and gRNAs are listed in **Table S2**.

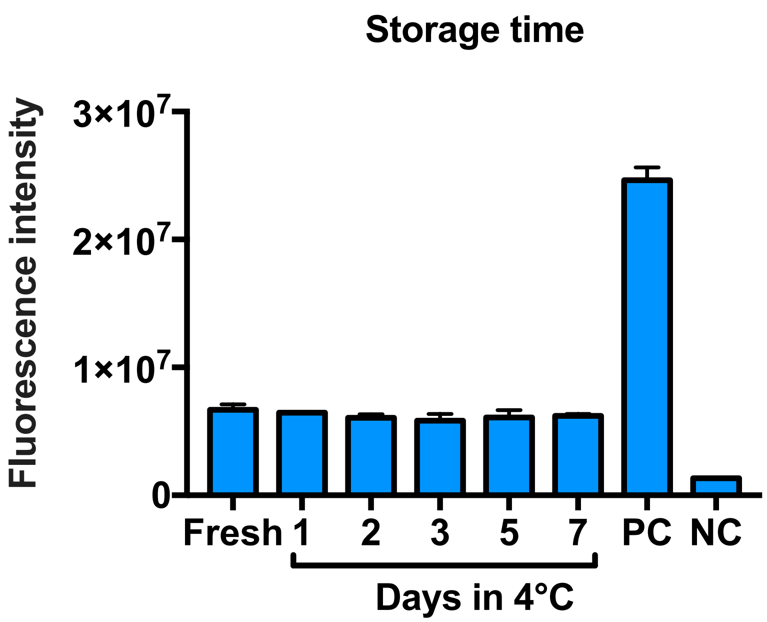

Figure S2. Effect of extended storage on SARS-Cov2 levels in spiked saliva samples.
Saliva samples from healthy human donors were spiked with 100 copies of heat-denatured SARS-Cov2, and RNA isolated from fresh or 4°C refrigerated samples was analyzed at the indicated time points. PC, positive control (100 copies heat-inactivated SARS-Cov2 in PBS), NC, negative control (PBS only).

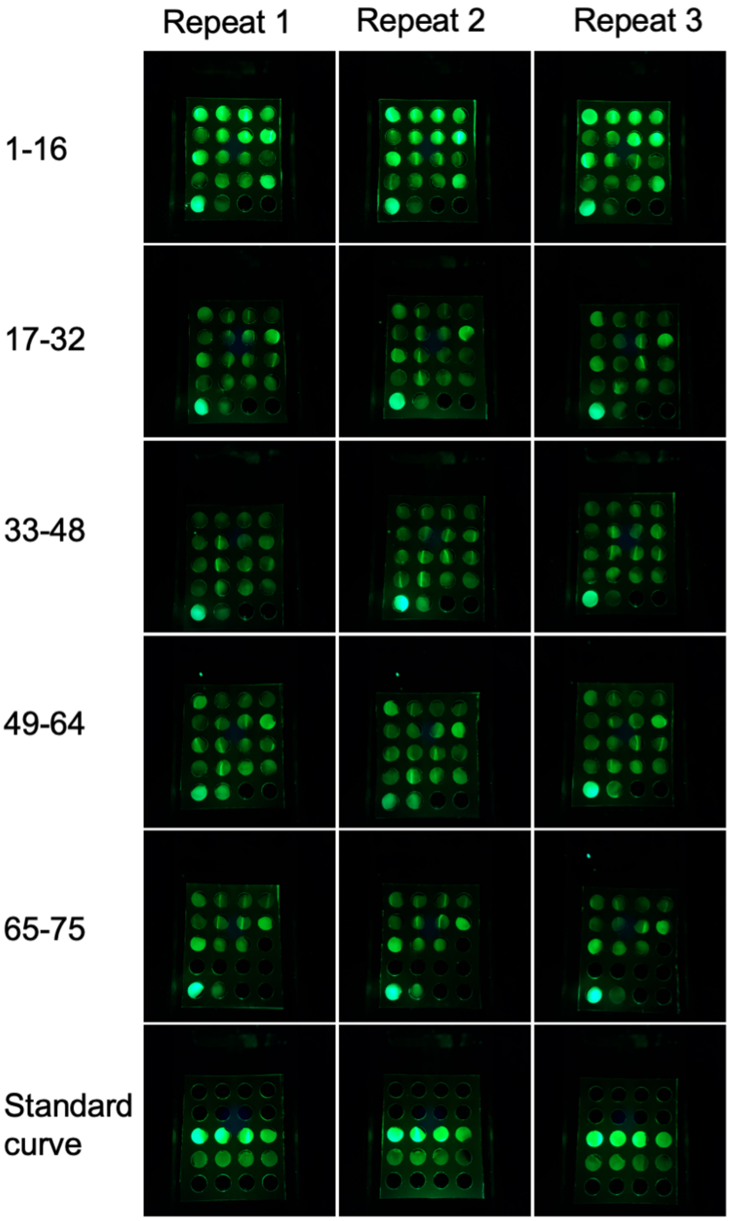

Figure S3. Repeatability of the saliva-based on-chip CRISPR-FDS smartphone assay.
75 patient saliva samples we analyzed by CRISPR-FDS smartphone assay with 3 times repeats. Positive control (10^2 copies synthetic SARS-CoV-2 RNA) and NC, Negative control was loaded in the bottom row of each chip. Standard curve was $1 \sim 10^5$ copy of copies synthetic SARS-CoV-2 RNA. Fluorescent signal image captured images captured with a 525nm filter with cellphone.
